# Demonstration of a Low-Cost and Portable Optical Cavity-Based Sensor through Refractive Index Measurements

**DOI:** 10.3390/s19092193

**Published:** 2019-05-12

**Authors:** Donggee Rho, Caitlyn Breaux, Seunghyun Kim

**Affiliations:** 1 Electrical Engineering Department, Baylor University, One Bear Place #97356, Waco, TX 76798, USA; donggee_rho@baylor.edu; 2 Biomedical Engineering Program, Baylor University, One Bear Place #97356, Waco, TX 76798, USA; caitlyn_breaux@baylor.edu

**Keywords:** optical cavity sensor, refractive index measurements, biosensors, portable system

## Abstract

An optical cavity-based sensor using a differential detection method has been proposed for point-of-care diagnostics. We developed a low-cost and portable optical cavity-based sensor system using a 3D printer and off-the-shelf optical components. In this paper, we demonstrate the sensing capability of the portable system through refractive index measurements. Fabricated optical cavity samples were tested using the portable system and compared to simulation results. A referencing technique and digital low pass filtering were applied to reduce the noise of the portable system. The measurement results match the simulation results well and show the improved linearity and sensitivity by employing the differential detection method. The limit of detection achieved was 1.73 × 10^−5^ Refractive Index Unit (RIU), which is comparable to other methods for refractive index sensing.

## 1. Introduction

For several leading causes of death worldwide, such as cancers, diabetes, respiratory diseases, and infectious diseases, early detection significantly increases survival rates by allowing timely initiation of treatment for patients [1]. However, current diagnostic technologies are mostly expensive and time consuming as they require centralized laboratories equipped with costly instruments operated by trained personnel [2]. For example, enzyme-linked immunosorbent assay (ELISA) is the gold standard diagnostic tool for detecting protein biomarkers with high sensitivity, but it requires lengthy and complicated procedures performed in a laboratory including multiple incubation, labeling, and washing steps [1,2]. With these issues, laboratory-based assays including ELISA are not appropriate for early diagnosis of diseases [3]. As an alternative to conventional diagnostic methods, a point-of-care (POC) device is emerging, which enables diagnostic tests at or near the patient’s bedside and eventually facilitates the detection and management of diseases at early stages [4]. There are commercially available POC diagnostic platforms based on lateral flow assays, which are used to diagnose pregnancy and HIV [5]. Even though the lateral flow test is attractive to be used in POC diagnostics, challenges still exist to test multiple analytes and have sensitive, quantitative, and reproducible test results [6,7]. Therefore, the development of a POC device that is low-cost, portable, user-friendly, robust, multiplexable, and sensitive is in high demand [8]. A successfully-implemented POC device would greatly improve public health [9], especially in developing countries with limited medical facilities.

An optical cavity-based sensor using a differential detection method has been developed for POC diagnostics [10,11,12,13,14,15,16]. The optical cavity consists of two partially reflective surfaces that are separated by a small gap. The transmission spectrum exhibits a resonance response. Receptor molecules are functionalized on the optical cavity surface, and a sample fluid is introduced to the optical cavity. As target biomarkers in the sample fluid are immobilized on the receptors, the refractive index inside the optical cavity increases, causing a shift in the resonance response. The conventional detection method requires the use of an expensive spectrometer or tunable laser source to measure the shift between the peaks. The proposed system measures the changing intensities at specific wavelengths using low-cost laser diodes and a CMOS camera to eliminate the need for expensive equipment. Besides that, the intensity-based detection method enables multiplexed assays. We proposed a differential detection method to equalize the measured intensity levels for each test, enhance the sensitivity, and improve fabrication tolerance [13]. As a proof of concept, refractive index measurements using the optical cavity samples have been performed on an optical table [14,15]. Its potential to be used as a sensitive, specific, and multiplexable biosensor has been also demonstrated through biotinylated Bovine serum albumin (BSA) detection using a streptavidin-coated surface with the system on an optical table [16]. The optical table not only offers a rigid surface to mount and align optical components readily but also helps to minimize the noise by isolating the optical system from ambient vibrations.

In this letter, we present a prototype of the portable and stand-alone optical cavity-based sensor and demonstrate its capability of detecting small refractive index changes. The portable system for the optical cavity-based sensor is described, and the simulation and measurement results are discussed.

## 2. Materials and Methods

### 2.1. Simulations

**Figure 1:**
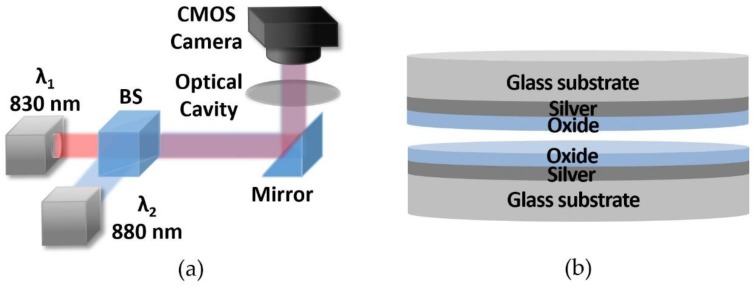
(**a**) Schematic diagram of the optical cavity-based sensor using 830 nm and 880 nm laser diodes. (**b**) Cross-sectional view of the optical cavity structure.

A schematic diagram of the optical cavity-based sensor is shown in Figure 1a. Two low-cost laser diodes at the wavelengths of 830 nm and 880 nm are used as light sources with collimators. The collimated laser beams are combined by a beam splitter, directed toward an optical cavity sample using a mirror, and propagate through the sample. A low-cost CMOS camera (Chameleon 3, FLIR, Wilsonville, OR, USA) is used as a detector at the end to measure light intensities of both wavelengths. Figure 1b shows a cross-sectional view of the optical cavity sample. Thin silver films on glass substrates act as partially reflective surfaces. Oxide layers provide adequate protection for the silver films against possible damages from fluid flow inside the optical cavity sample and facilitates the functionalization process for biosensing applications [16].

**Figure 2:**
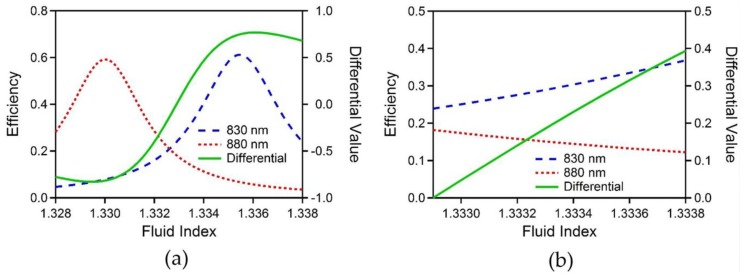
(**a**) Simulation results showing efficiencies of 830 nm (blue dashed line) and 880 nm (red dotted line) and differential value (green solid line) versus the refractive index inside the optical cavity in the range between 1.328 and 1.338. (**b**) Simulation results as shown in Figure 2a with the range of refractive index between 1.3329 and 1.3338.

The simulations were conducted using FIMMWAVE/FIMMPROP (Photon Design). The simulation results for the optical cavity structure for the refractive index range between 1.328–1.338 are shown in Figure 2a. Along with the transmission efficiencies at the wavelengths of 830 nm and 880 nm, the differential value (η) is calculated by the equation below mainly to enhance the sensitivity and linearity.

(1) $$\eta =\frac{I_{1}-I_{2}\frac{I_{10}}{I_{20}}}{I_{1}+I_{2}\frac{I_{10}}{I_{20}}}$$

$I_{1}$ and $I_{2}$ are the optical intensities (or efficiencies for simulations) at 830 nm and 880 nm, respectively, and $I_{10}$ and $I_{20}$ are the initial values of $I_{1}$ and $I_{2}$, respectively.

The optical cavity structure is designed to have a high sensitivity and linearity near the refractive index of 1.333, which is close to the refractive index of biological fluids for biosensing purposes [17]. The design parameters are a cavity width (distance between silver layers) of 10.14 µm, a silver thickness of 18 nm, and an oxide thickness of 400 nm. As seen in Figure 2a, the efficiencies of 830 nm and 880 nm show resonance responses within that range with an offset. Due to this offset, the efficiency of 880 nm decreases while the efficiency of 830 nm increases over the refractive index range of our interest that includes 1.333. Figure 2b shows the simulation results within the refractive index range from 1.3329 to 1.3338. In this range, the efficiency of 830 nm has a slope of 143.91 /RIU with an R^2^ of 0.996, and the efficiency of 880 nm has a slope of −66.19 /RIU with an R^2^ of 0.9942. As the efficiencies change in opposite directions, the differential value has a higher slope of 439.31 /RIU and shows better linearity with an R^2^ of 0.999 compared to the individual wavelength efficiency.

### 2.2. Portable Systemix

**Figure 3:**
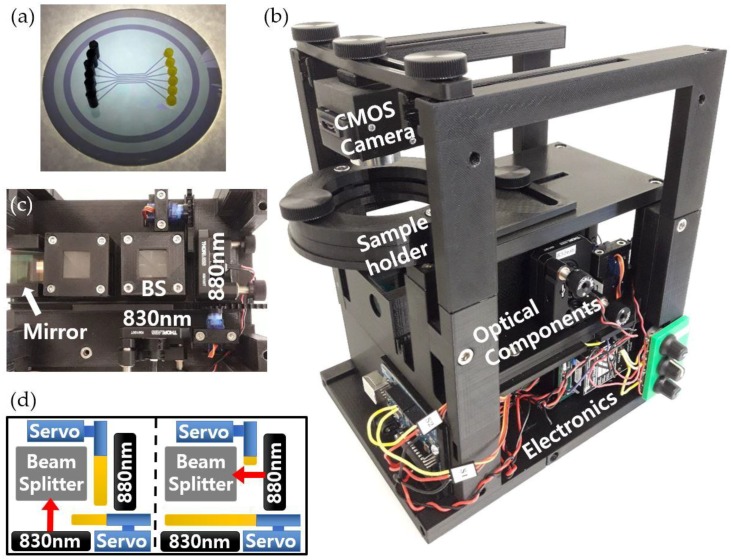
(**a**) Fabricated optical cavity sample including 6 fluidic channels. 3D printed adapters are attached to inlets and outlets. (**b**) Prototype of portable optical cavity-based sensor. (**c**) Optical components mounted on the middle level plate of the portable system. (**d**) Schematic of servo motors (blue parts) with blocking plates (yellow parts) to block laser diodes alternately.

Figure 3a shows a fabricated optical cavity sample including 6 fluidic channels. The fabrication process was very simple, using two 3-inch glass substrates. A glass substrate was drilled with a 1 mm diamond drill bit to make inlets and outlets. Thin silver films were deposited on both glass substrates by sputtering. The silver film on a drilled glass substrate was patterned and etched to have isolated silver patterns along the fluidic channels. Subsequently, the oxide layer was formed on both substrates with spin-on-glass (SOG) and silicon dioxide (SiO_2_) layers. SOG layers were spin-coated at 1400 Revolutions Per Minute (RPM) and then cured on a hot plate (PC-400D, Corning, Corning, NY, USA) at 130 °C for 4 min. The fluidic channels were defined by a photolithography process using SU8 2010 (Microchem, Westborough, MA, USA) on an undrilled substrate. The two glass substrates were then bonded by applying a thin layer of UV glue onto the SU8 structure and curing in a UV-box for 30 s. The fluidic channel width and length were 550 µm and 36 mm, respectively, and the total sample volume required to fill the entire channel was 1.1 µL. Note that the fabrication processes were adjusted until we achieved measurement results similar to the simulation.

The optical cavity sample with 3D printed input and output adapters attached is placed on the sample holder of the portable system shown in Figure 3b. A prototype of the portable system for the optical cavity-based sensor was fabricated using a 3D printer (Creator Pro, FLASHFORGEUSA, City of Industry, CA, USA), which enables rapid materialization of the design and allows the system to be upgraded efficiently. All structural components, excluding hardware and certain optical mounts, were fabricated via fused deposition modeling with polylactic acid (PLA). The system was designed in tiers that were assembled after printing the parts. The bottom level contains the electrical components including a laser diode driver, an Arduino for servo motor control, and a multi-channeled output power supply with its switch. The middle level houses the optical components including 830 nm and 880 nm laser diodes with collimators, kinematic mounts, two servo motors, a 50:50 beam splitter, and a right-angled mirror as shown in Figure 3c. In order to measure the intensities of laser diodes and obtain differential values in real-time using one camera, two servo motors are used to alternately block the light from one laser diode at a time with one-second intervals. The servo horns are affixed with gears, and the servo motors rotate to move 3D-printed gear rack blocking plates back and forth as shown in Figure 3d. The top level consists of the sample holder, a plate fixed to the sample holder below, and the CMOS camera fixed to a camera mount. For precise and simple optical alignment, the parts mounted in the top level are designed to be more adjustable with thumbscrews. The overall dimensions for the portable system are approximately 6.5 inches wide by 8.5 inches in length by 11 inches in height, weighing no more than 10 lbs. All parts in the system can be bought off-the-shelf, and the total cost to build is about $ 1500.

## 3. Results and Discussion

The refractive index measurements using the portable system were performed on a table with wheels to demonstrate the sensitivity of the portable system in typical user settings. The fluidic channel of an optical cavity sample was aligned with the center of the collimated laser beams, and the CMOS camera continuously captured images of beam profiles to measure laser intensities in real-time. A 40 × 40 pixel array of the CMOS images was selected near the middle of the channel to calculate the average pixel intensity. Based on the size of a pixel, the chosen pixel area is approximately equal to the area of 140 µm × 140 µm.

Deionized (DI) water (Refractive Index (RI): 1.3329) and Dimethyl sulfoxide (DMSO) (RI: 1.4787) are mixed with different ratios to produce 5 refractive index fluids, 1.3329, 1.3332, 1.3334, 1.3336, and 1.3338, which were confirmed with a digital refractometer (PA202, MISCO, Solon, OH, USA). The channel was initially filled with DI water to acquire baseline intensities for 20 s. DI water in the channel was then replaced with a refractive index fluid of 1.3332. After another 20 s, DI water was again introduced to the channel to recover the baseline. The same procedure was then repeated for the rest of three refractive index fluids to calculate the changes in both intensities from the baseline signal. During measurements, the portable system was covered with an enclosure to avoid ambient light reaching the CMOS camera. Throughout the measurement, the volume of each fluid introduced was 2 µL, and the fluids were controlled by a mini vacuum pump connected to an output port via Tygon tubing. The entire time spent measuring five refractive index fluids was about 8 min.

Since the refractive index measurements were performed on a relatively unstable table, the measured intensities were noisier than those with an optical table. One of the major noise sources may be the vibrations introduced to the portable system from the internal parts and surroundings. Such noise due to the vibration could be common among the measured channel and other areas around it including adjacent, unused empty channels. To effectively cancel out common variations, we employed a referencing technique [10]. The referencing technique is designed to remove common variations from the measured data using the reference data collected from the adjacent empty channel using the equation below.

(2) $$I_{P}=I_{D}-I_{R}\frac{I_{D_{0}}}{I_{R_{0}}}+I_{D_{0}}$$

**Figure 4:**
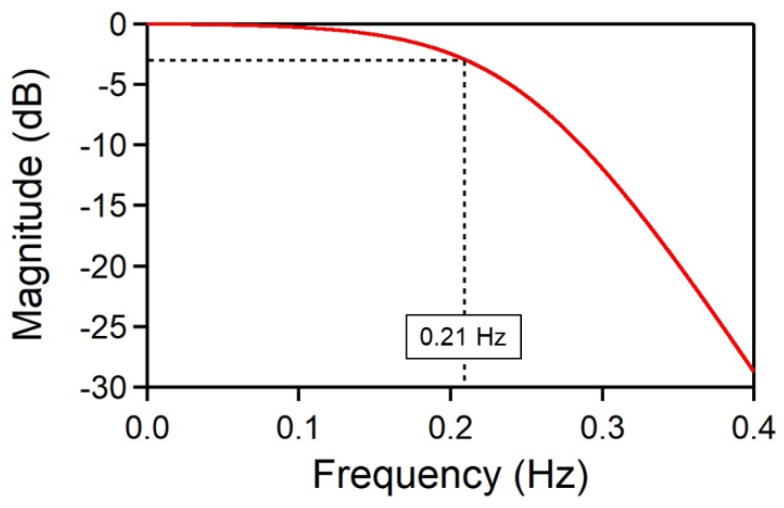
The frequency response of the digital low-pass filter (LPF).

$I_{P}$ is the processed average pixel intensity, $I_{D}$ and $I_{R}$ are the average intensity measured at the tested channel and the average intensity measured at the empty channel (as a reference), respectively. $I_{D_{0}}$ and $I_{R_{0}}$ are the initial values of $I_{D}$ and $I_{R}$, respectively. The collimated beam and CMOS imaging area are large enough to collect the data from multiple channels including the test channel and adjacent one. To lower the noise level further, a low-pass filter (LPF) was applied digitally to the processed intensities. The frequency response of the digital LPF is shown in Figure 4. Table 1 shows that the standard deviation of the differential values decreases by about 70% as we applied the referencing technique and LPF to the raw data.

**Figure 5:**
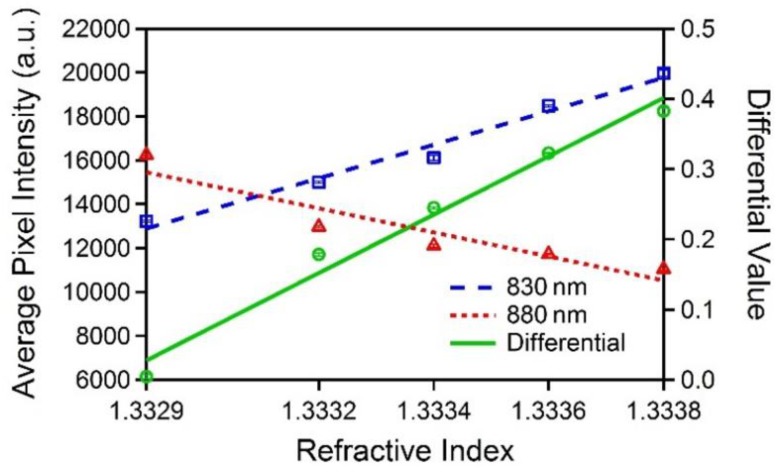
Measurement results showing the average pixel intensities for 830 nm (blue dashed line) and 880 nm (red dotted line) and differential value (green solid line) versus the refractive indices in the same range (1.3329–1.3338) as shown in Figure 2b.

Figure 5 shows the measurement results. The average pixel intensities of two laser diodes and the calculated differential values are shown in the figure with their respective linear fits as a function of the refractive index fluids. The error bars indicate +/− standard deviation of each data point, but they are too small to be visible.

As the refractive index increases from 1.3329 to 1.3338, the average intensity of 830 nm increases from 13,223 to 19,972, and the average intensity of 880 nm decreases from 16,253 to 11,051. The corresponding differential value is increased from 0 to 0.383 with a slope of 415.1 /RIU. The measured differential value slope is close to the slope of 439.31 /RIU obtained in the simulation.

Based on the measurement results, the limit of detection (LOD) for 830 nm, 880 nm, and the differential value are estimated using the slope and standard deviation (LOD = 3σ / S, where σ is the standard deviation and S is the slope). The standard deviation is obtained by taking an average of five standard deviations at each refractive index fluid. Table 2 shows the compared LOD for 830 nm, 880 nm, and the differential value along with R^2^ values and the relative standard deviations (RSDs). The LOD of the differential value was 1.73 × 10^−5^ RIU which is 45% and 37% better than that of 830 nm and 880 nm, respectively. This LOD is comparable with other methods reported that are neither low-cost nor portable [18,19,20,21,22,23,24].

As shown in Table 2, the R^2^ value of the differential values is also better than that of individual wavelengths. Therefore, the measurement results confirm that the differential calculation method enhances the sensitivity and linearity compared to individual wavelengths as expected. To evaluate the results more in depth, we calculated RSDs to directly compare noises of each wavelength and the differential values using the average value and standard deviation (RSD = σ / n × 100 (%), where σ is the standard deviation and n is the average value) [25]. As shown in Table 2, the RSD of the differential value is increased compared to each wavelength which indicates the noise in the differential values is larger than that of individual wavelengths. This explains why the improvement on LOD for the differential values with respect to individual wavelengths is smaller than the improvement on the slopes from the simulations. Based on these results, we can conclude that the differential value by taking the differential calculation of two intensities changing in opposite directions increases the magnitude of change, however, it also increases noise which in turn limits the improvement on LOD. At the same time, this also indicates that we could improve LOD further by lowering the noise in differential values. One possible solution for this is to employ the simultaneous detection method rather than the sequential detection method we used to effectively reduce the common variations existing in both measured intensities.

## 4. Conclusions

In conclusion, we presented a prototype of a portable optical cavity-based sensor and performed refractive index measurements to demonstrate its capability as a stand-alone system. The optical cavity structure is designed to have a cavity width of 10.14 µm, a silver thickness of 18 nm, and an oxide thickness of 400 nm. The simulation results showed that the differential value has an increased slope with a better linearity compared to the individual efficiency changes. The prototype of this portable system was built using a 3D printer and incorporated off-the-shelf optical components. The optical cavity sample was fabricated through simple microfabrication processes, and refractive index measurements were performed using the portable system. The noise level was decreased by about 70% by using the referencing technique and applying a digital low-pass filter. The slope of the differential values from the measurement was 415.1 /RIU, which is close to the simulation results. The LOD of the portable optical cavity-based sensor for refractive index sensing was calculated to be 1.73 × 10^−5^ RIU providing the potential for high sensitivity biosensing applications. Detailed analysis also revealed that the LOD can be improved further by reducing the noise in the differential values.

## Figures and Tables

**Table 1:** The standard deviation (STD) by applying the referencing technique and the low-pass filter (LPF) compared to that of the raw data.

Methods	STD
Raw data	8.49 × 10^−3^
Apply Equation (2)	3.35 × 10^−3^
Apply Equation (2) and LPF	2.29 × 10^−3^

**Table 2:** The limit-of-detection (LOD), R^2^, and relative standard deviations (RSD) for 830 nm, 880 nm, and differential value obtained by the refractive index measurement.

	LOD (RIU)	R^2^	RSD (%)
830 nm	3.12 × 10^−5^	0.9796	0.48
880 nm	2.73 × 10^−5^	0.8803	0.39
Differential	1.73 × 10^−5^	0.9802	1.05
